# Keratinolytic proteases secreted by *Bacillus subtilis* obtained from feather-based submerged culture

**DOI:** 10.1007/s42770-026-01993-x

**Published:** 2026-06-18

**Authors:** Cíntia Lionela Ambrósio de Menezes, Rafael Amadeu Barreto, Emanuella Roberto Ribeiro, Érike Jhonnathan Pereira, Luana De Lucca Thomaz, Maurício Boscolo, Roberto da Silva, Eleni Gomes, Ronivaldo Rodrigues da Silva

**Affiliations:** Instituto de Biociências, Letras e Ciências Exatas, Universidade Estadual Paulista “Júlio de Mesquita Filho” – São José do Rio Preto, R/ Cristóvão Colombo, 2265. Jd Nazareth, Ibilce-Unesp, São Paulo, Brazil

**Keywords:** Feather, Keratin, Keratinases, Peptidases, Proteases

## Abstract

Microbial keratin degradation has been recognized as a sustainable strategy for treating keratin-rich residues, as it avoids the use of environmentally harmful chemical agents. The present study aimed to evaluate the ability of *Bacillus subtilis* to degrade chicken feathers and to characterize the proteolytic enzymes it secretes. The enzymes were produced by *B. subtilis* in submerged fermentation, using chicken feathers (0.5% w/v) as the sole source of carbon and nitrogen. The resulting enzymatic extract contained multiple proteases, as evidenced by their detection in polyacrylamide gels copolymerized with gelatin. This extract exhibited distinct optimal pH ranges for keratinolytic and caseinolytic activities. Inhibition assays revealed that keratinolytic activity was strongly affected by phenylmethylsulfonyl fluoride (PMSF), with reductions of 49.3% at pH 7.0 and 88.4% at pH 9.5, and by ethylenediaminetetraacetic acid (EDTA), which caused inhibitions of 69.2% and 78.7% at pH 7.0 and 9.5, respectively. Caseinolytic activity was even more sensitive to these inhibitors, particularly at pH 9.5. Enzymes exhibiting higher activity at pH 7.0 displayed a greater capacity to hydrolyze native keratinous substrates compared with those active under alkaline conditions. Overall, these findings highlight *B. subtilis* as a promising source of keratinolytic enzymes with favorable biochemical properties for biotechnological applications, including biofilm dispersion and production of bioactive peptides.

**Supplementary Information:**

The online version contains supplementary material available at 10.1007/s42770-026-01993-x.

## Introduction

Feathers constitute a primary by-product in the poultry industry, comprising 5%–10% of chicken body weight [1–3]. On a dry weight basis, feathers are nearly 90% keratin, an insoluble, compact fibrous protein rich in cysteine. Keratin polypeptide chain structures are stabilized by disulfide bonds, hydrophobic interactions, and hydrogen bonding, resulting in rigid and highly ordered molecular structures. Such structural complexity imbues keratins with excellent mechanical properties including resistance to mechanical stress and to many proteases including papain, pepsin, and trypsin [1, 4, 5].

The global poultry industry produces millions of metric tons of feather waste annually, negatively impacting the environment and waste management because feathers are disposed of inefficiently in landfills or through incineration, and limited reuse strategies, such as feather meal, currently exist [6]. Alternative feather degradation strategies rely on chemical hydrolysis, frequently requiring harsh conditions and producing environmentally undesirable by-products (highly concentrated acidic or alkaline solutions). Enzymatic hydrolysis represents a more renewable option with better capacity to degrade keratin under mild conditions that reduce environmental load [7].

Keratinases are proteolytic enzymes that can degrade keratinous substrates, including feathers, hair, nails, and wool. These enzymes exhibit substantial diversity in their biochemical and biophysical properties, including distinct pH and temperature ranges for activity and stability, and variations in cofactor dependence and substrate specificity [6, 8].

Keratinolytic microorganisms can solubilize feather waste during submerged fermentation, producing a fermentative extract rich in proteases and keratin-derived peptides. Keratin hydrolysates may exhibit antioxidant and metal-chelating properties and can be tested as biofertilizers, animal feed supplements, and substrates for biogas production. Keratinases have been investigated for applications in biofilm dispersion, enhancement of nail permeability for onychomycosis treatment, and the development of cosmetic formulations for skin and hair care[6, 9, 10].

To maximize the feasibility of feather waste reclamation to produce value-added products, increased efforts should be concentrated on studies of keratinase producing microorganisms and detailed characterization of their enzymes. In this study, we evaluated the ability of *Bacillus subtilis* to produce keratinases during submerged fermentation using chicken feathers as the sole keratin source, and we performed a biochemical characterization of the resulting enzyme extract. Given the versatility of keratinases in hydrolyzing various proteins, this method could serve as a useful starting point for the disruption of biofilms and the production of bioactive peptides in future studies.

## Materials and methods

### Microorganism and submerged fermentation

The *B. subtilis* strain used in this study was kindly provided by the Laboratory of Biochemistry and Applied Microbiology, São Paulo State University (UNESP), São José do Rio Preto, São Paulo, Brazil. Stock cultures were maintained at − 80 °C in 15% (v/v) glycerol (final concentration).

For pre-culture preparation, cells were recovered from the glycerol stock, streaked onto Luria–Bertani (LB) agar plates (10 g L^− 1^ sodium chloride, 10 g L^− 1^ Tryptone, 5 g L^− 1^ yeast extract, and 15 g L^− 1^ agar), and incubated at 30 °C for 12 h. A single colony was then inoculated into 100 mL of LB broth in a 250 mL Erlenmeyer flask and cultivated under shaking conditions to obtain a bacterial suspension (30 °C and 150 rpm for 16 h).

Keratinase production was performed by submerged fermentation. The fermentation medium consisted of 0.7 g L^− 1^ KH_2_PO_4_, 1.5 g L^− 1^ K_2_HPO_4_, 0.1 g L^− 1^ MgSO_4_, 0.5 g L^− 1^ NaCl, and 5 g L^− 1^ chicken feathers as the sole carbon and nitrogen source. The initial pH was adjusted to 7.2, following the protocol described by Duffeck et al. [11]. The medium was inoculated with 5.0 mg of dry bacterial biomass per 50 mL of liquid medium and incubated at 30 °C with agitation at 150 rpm for 48 h (Online Resource 1).

After fermentation, cultures were filtered through Whatman No. 1 filter paper and centrifuged at 10,000 × *g* for 10 min at 4 °C to remove residual solids and cells. The resulting cell-free supernatant was concentrated by tangential flow filtration using a hollow fiber membrane with a 10 kDa molecular weight cutoff (surface area = 420 cm^2^; model UFP-10-E-4MA, GE Healthcare).

## Determination of keratinolytic and caseinolytic activities

Keratinolytic and caseinolytic activities of the fermentative extract were determined using keratin azure (Sigma-Aldrich) and bovine casein (Sigma-Aldrich) as substrates, respectively. All assays were performed in triplicate.

For the keratinolytic activity assay, 200 µL of the fermentative extract was incubated with 1 mL of 0.2 M Tris–HCl buffer (pH 8.0) containing 0.01 g of keratin azure. The reaction mixture was maintained at 45 °C with agitation at 180 rpm for 3 h. Enzymatic activity was terminated by the addition of 600 µL of 10% (w/v) trichloroacetic acid (TCA). The mixture was then centrifuged, and the absorbance of the supernatant was measured at 595 nm using a spectrophotometer. A control assay, in which 10% TCA was added to the enzyme solution prior to substrate addition, was included in all experiments. One unit (U) of keratinolytic activity was defined as the amount of enzyme required to increase absorbance by 0.01 at 595 nm per hour under the assay conditions [12, 13].

Caseinolytic activity was determined by mixing 50 µL of the fermentative extract with 500 µL of a 1% (w/v) casein solution prepared in 0.2 M Tris–HCl buffer (pH 8.0). The reaction mixture was incubated at 45 °C for 10 min and subsequently stopped by the addition of 300 µL of 10% TCA. After centrifugation at 10,000 × *g* for 10 min at 25 °C, the absorbance of the supernatant was measured at 280 nm against a control in which TCA was added prior to substrate addition. One unit (U) of caseinolytic activity was defined as the amount of enzyme required to increase absorbance by 0.01 at 280 nm per minute under the assay conditions [14].

## Effect of pH, temperature, inhibitors, and thermal stability on enzyme activity

The effect of pH on enzyme activity was evaluated at 45 °C using the following 0.2 M buffer systems: acetate (pH 5.5), MES (pH 6.0 and 6.5), HEPES (pH 7.0 and 7.5), BICINE (pH 8.0 and 8.5), glycine (pH 9.0 and 9.5), and CAPS (pH 10.0, 10.5, and 11.0). The effect of temperature on enzymatic activity was assessed over a range of 30–65 °C, in increments of 5 °C. Relative activity was calculated by defining the highest activity observed under the tested conditions as 100%.

Thermal stability was evaluated by pre-incubating the enzyme extract for 1 h at temperatures ranging from 30 to 60 °C. After pre-incubation, enzymatic activity was measured under the previously established optimal pH and temperature conditions. Enzyme activity without pre-incubation was considered 100% and used as the reference for relative stability calculations.

To investigate the catalytic nature of the proteases, the inhibitors phenylmethylsulfonyl fluoride (PMSF), ethylenediaminetetraacetic acid (EDTA), *N*-ethylmaleimide (NEM), and iodoacetic acid (IAA) were evaluated at a final concentration of 5 mM. Enzyme preparations were pre-incubated with each inhibitor for 5 min at the reaction temperature prior to substrate addition. All assays were conducted under the predetermined optimal pH and temperature conditions. Enzyme activity measured in the absence of inhibitors was defined as 100%.

## Proteolytic activity on electrophoresis gel

Native SDS–PAGE zymography was performed using 8% polyacrylamide gels copolymerized with 0.2% gelatin as substrate. Protein samples were mixed with loading buffer lacking reducing agents (β-mercaptoethanol) and were not subjected to thermal denaturation prior to electrophoresis. The effect of protease inhibitors was assessed by incubating the sample with each inhibitor, as described in the previous section, at a final concentration of 5 mM. Control reactions without inhibitors and with thermally denatured samples were included.

Following electrophoresis, gels were incubated in 2% Triton X-100 for 30 min to remove SDS, and subsequently washed with 0.1 M Tris–HCl buffer (pH 8.0). Gels were then incubated in the same buffer for 2 h at 45 °C to allow substrate digestion. After incubation, the buffer was removed and the gels were stained with 0.2% Coomassie Brilliant Blue R-250 for 4 h, followed by destaining in a solution containing 12% acetic acid and 50% methanol. Clear, unstained bands on the gel were interpreted as regions of proteolytic activity.

## Effect of metal ions on enzyme activity

The influence of metal ions on enzymatic activity was assessed by incubating the enzyme with each chloride salt at a final concentration of 5 mM. The following metal ions were evaluated: barium (BaCl_2_), calcium (CaCl_2_), cobalt (CoCl_2_), copper (CuCl_2_), iron (III) (FeCl_3_), lithium (LiCl), magnesium (MgCl_2_), manganese (MnCl_2_), mercury (HgCl_2_), potassium (KCl), and sodium (NaCl). For all assays, the fermentative extract was pre-incubated with each chloride salt for 5 min at room temperature. Subsequently, enzymatic reactions were performed under the previously determined optimal pH and temperature conditions.

### Proteolytic activity on different keratin substrates

Proteolytic activity toward distinct keratin sources was assessed using 0.01 g of each substrate (white chicken feather, sheep wool, cow horn, and keratin azure) incubated with 200 µL of fermentative extract in 1 mL of either HEPES buffer (0.2 M, pH 7.0) or glycine buffer (0.2 M, pH 9.5). Reaction mixtures were maintained at 45 °C for 15 h under agitation at 180 rpm. Enzymatic reactions were subsequently terminated by the addition of 600 µL of 10% TCA. The samples were centrifuged, and the resulting supernatants were used to quantify proteolytic activity by measuring absorbance at 280 nm (white feather, sheep wool, and cow horn substrates) or at 595 nm (keratin azure) against their respective controls. Control reactions consisted of fermentative extract to which 10% TCA was added prior to substrate addition. All assays were performed in triplicate.

### Data analysis

All measurements were conducted using a minimum of three independent replicates. Experimental results are reported as mean values accompanied by their respective standard deviations (mean ± SD).

Statistical significance was evaluated using one-way analysis of variance (ANOVA) followed by Dunnett’s post hoc test for multiple comparisons. Differences were considered statistically significant at *p* ≤ 0.05. All statistical analyses were performed using IBM SPSS Statistics v.20 (IBM Corp., Armonk, NY, USA) and GraphPad Prism v.9 (GraphPad Software, San Diego, CA, USA).

## Results

### Effects of pH and temperature on enzyme activity and thermal stability

**Fig. 1:**
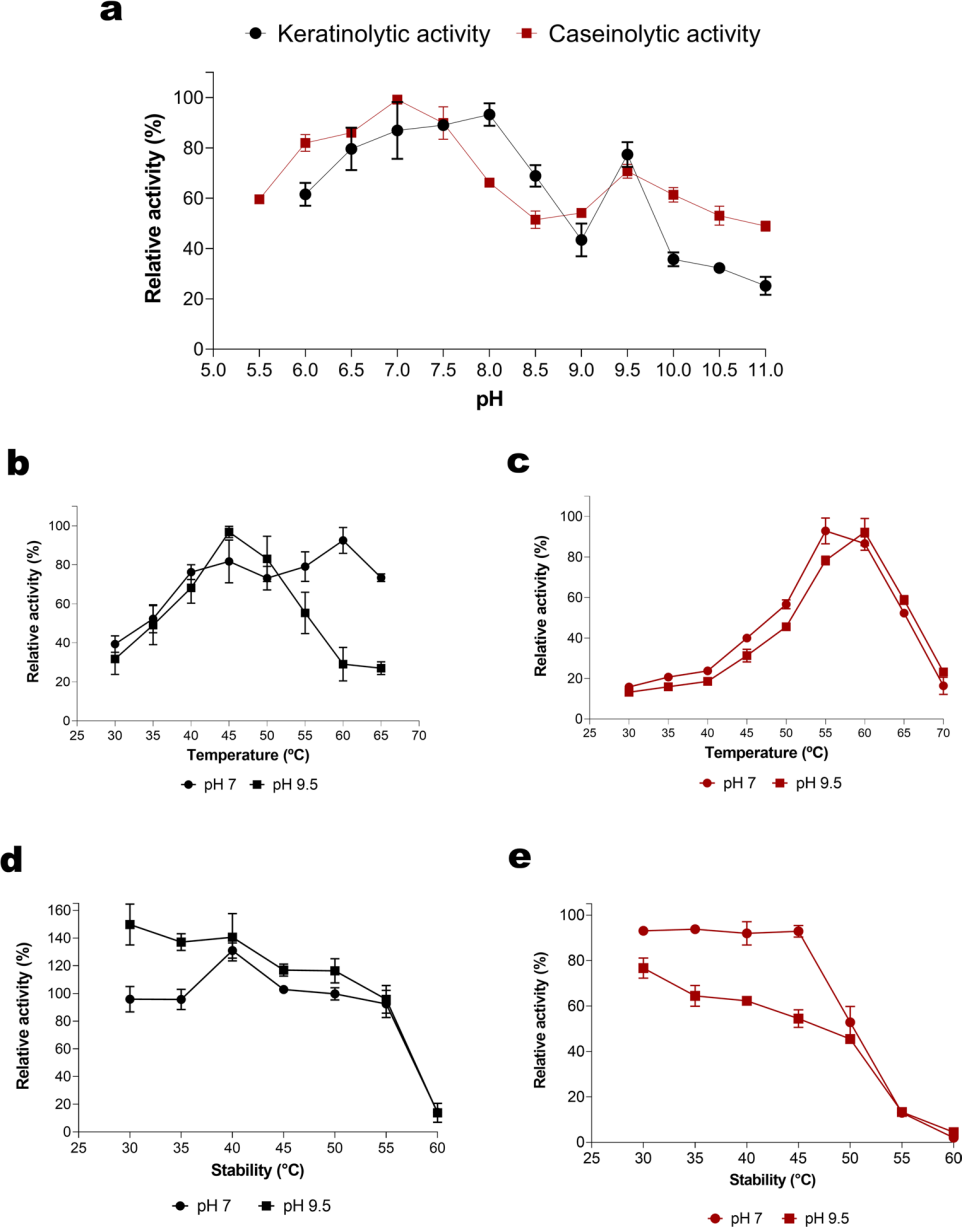
Characterization of the *B. subtilis* fermentative extract. Effect of pH on keratinolytic and caseinolytic activities (**a**); effect of temperature on keratinolytic (**b**) and caseinolytic (**c**) activities; and thermal stability of the enzymes, assessed by keratinolytic (**d**) and caseinolytic (**e**) activities. Black lines represent keratinolytic activity, and red lines represent caseinolytic activity. Data are presented as mean ± SD (*n* = 3). Statistical significance was assessed by one-way ANOVA/Dunnett’s test (*p* < 0.05)

Keratinases were produced by *B. subtilis* cultivated in liquid medium containing chicken feathers as its sole carbon and nitrogen source. The fermentative extract exhibited maximal keratinolytic activity within two distinct pH intervals (Fig. 1a). Between pH 6.5 and 8.0, no statistically significant differences were detected (*p* > 0.05), with the enzymes displaying more than 75% relative activity. A gradual decline was observed from pH 8.5–9.0, followed by a pronounced activity peak (77.38%) at pH 9.5. A similar trend was noted in the caseinolytic assay, in which relative activity exceeded 80% between pH 6.0 and 7.5, with maximal activity detected in the pH 7.0–7.5 range (*p* > 0.05). Activity decreased thereafter, but again spiked to a secondary maximum of approximately 70.7% at pH 9.5.

The effects of temperature on enzymatic activity were thus evaluated at pH 7.0 and 9.5. At pH 7.0, maximal keratinolytic activity (Fig. 1b) occurred between 45 and 60 °C, whereas at pH 9.5, peak activity occurred within a narrower range of 45–50 °C (*p* > 0.05). For caseinolytic activity (Fig. 1c), maximum activities were observed at 55 and 60 °C (*p* > 0.05) at pH 7.0 and at 60 °C at pH 9.5.

Keratinases demonstrated substantial thermal stability (Fig. 1d), maintaining > 80% residual activity after 1 h of incubation at temperatures up to 55 °C at both pH values. For caseinolytic activity (Fig. 1e), proteases displayed higher stability at pH 7.0, retaining 92.9% residual activity up to 45 °C, whereas at pH 9.5, residual activity decreased to 54.45% at 45 °C.

### Effects of protease inhibitors on enzymatic activity

**Fig. 2:**
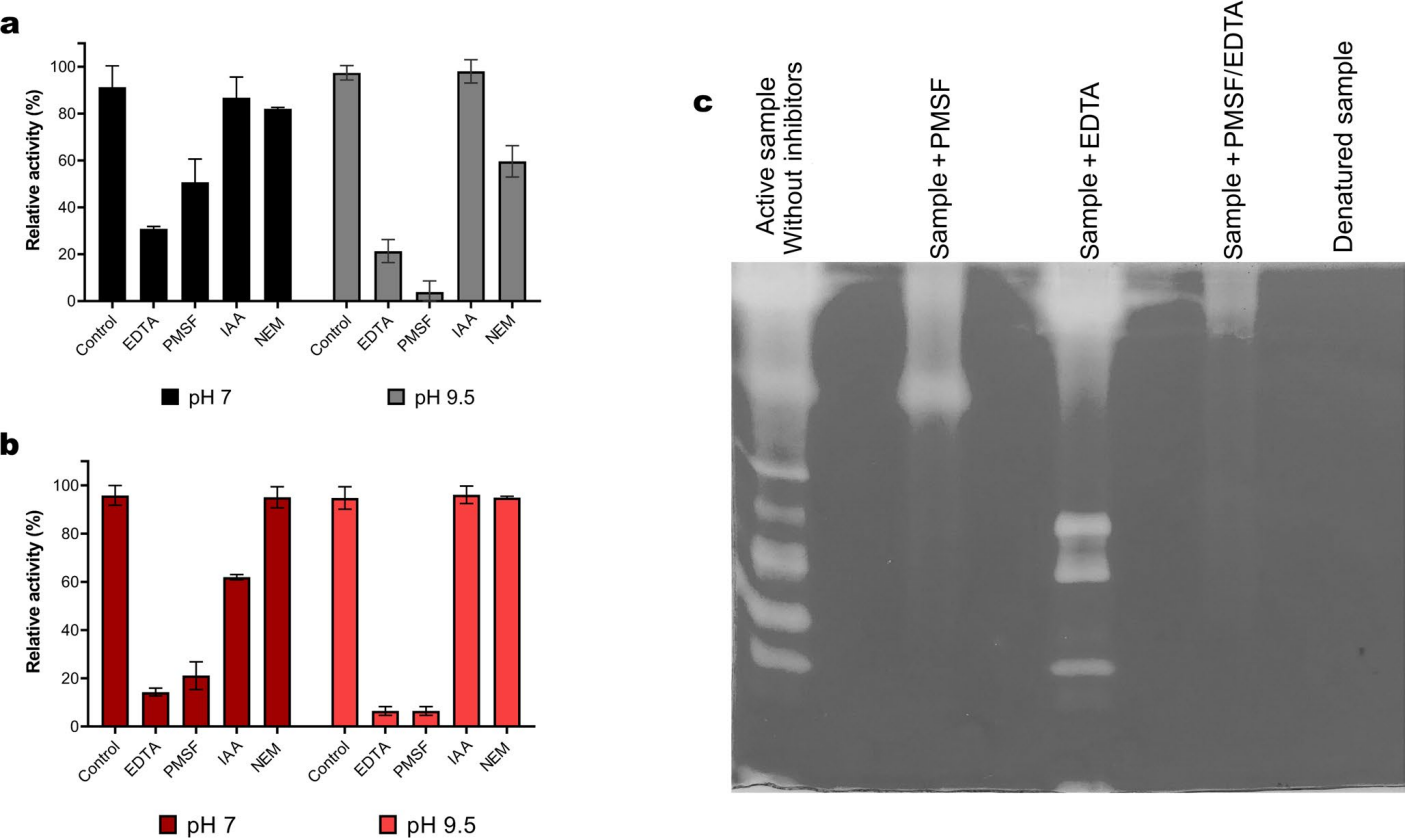
Effect of protease inhibitors (5 mM) on keratinolytic (**a**) and caseinolytic (**b**) activities at different pH values. The inhibitors ethylenediaminetetraacetic acid (EDTA), phenylmethylsulfonyl fluoride (PMSF), iodoacetic acid (IAA), and *N*-ethylmaleimide (NEM) were evaluated. Zymographic analysis performed on an 8% polyacrylamide gel copolymerized with 0.2% gelatin, illustrating the effects of EDTA and PMSF on protease activity from the *B. subtilis* enzymatic extract (**c**). Data are presented as mean ± SD (*n* = 3). Statistical significance was assessed by one-way ANOVA/Dunnett’s test (*p* < 0.05)

Incubation of the fermentative extract with 5 mM protease inhibitors resulted in pronounced inhibition of enzymatic activity. Keratinolytic activity (Fig. 2a) was significantly reduced by PMSF at both pH values, with decreases of 49.3% at pH 7.0 and 88.4% at pH 9.5. EDTA also markedly inhibited keratinolytic activity, causing reductions of 69.2% (pH 7.0) and 78.7% (pH 9.5). Similar inhibition patterns were observed for caseinolytic activity (Fig. 2b), which was particularly affected at pH 9.5, where both PMSF and EDTA promoted a 96% decrease in activity.

Native SDS-PAGE analyses (Fig. 2c) confirmed the inhibitory effects of PMSF and EDTA, revealing at least seven bands with collagenolytic activity. Smaller proteases were strongly inhibited by PMSF, whereas apparently one band was specifically inhibited by EDTA. The combined presence of both inhibitors nearly abolished all detectable proteolytic activity in the extract, indicating that the majority of enzymes present are serine proteases and metalloproteases.

### Interference of metal ions on enzyme activity

**Fig. 3:**
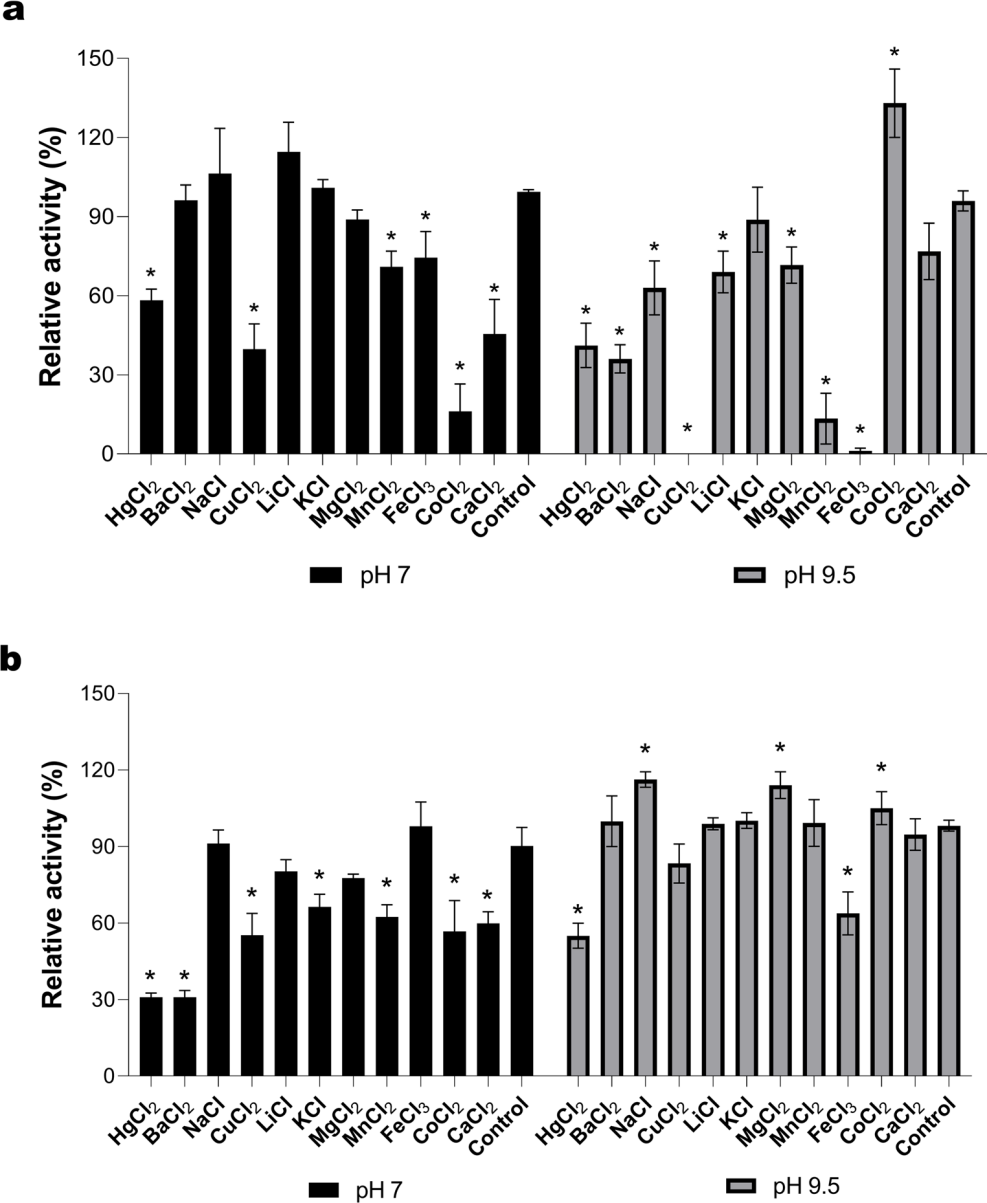
Interference of metal ions (5 mM) on keratinolytic (**a**) and caseinolytic (**b**) activities at different pH. Data are presented as mean ± SD (*n* = 3). Statistical significance was assessed by one-way ANOVA/Dunnett’s test (*p* < 0.05), and significant differences are indicated by an asterisk (*)

Keratinolytic activity at pH 7.0 was not enhanced by any of the evaluated metal ions (no statistically significant differences were observed relative to the control). In contrast, Ca^2+^, Co^2+^, Cu^2+^, Fe^3+^, Hg^2+^, and Mn^2+^ significantly decreased enzymatic activity, with Co^2+^ showing the largest effect (83.8% reduction in catalytic performance) (Fig. 3a).

At pH 9.5, Co^2+^ yielded the strongest positive effect, enhancing keratinolytic activity by 33%. Conversely, Cu^2+^ caused complete loss of enzymatic activity at this pH, and Fe^3+^ and Mn^2+^ respectively reduced activity by 98.8% and 86.6%. Ba^2+^, Hg^2+^, Li^+^, Mg^2+^, and Na^+^ also negatively modulated keratinolytic activity (Fig. 3a).

Most tested metals negatively modulated caseinolytic activity at pH 7.0, with activity reductions of 69% for Ba^2+^ and Hg^2+^, 44.75% for Cu^2+^, 43.25% for Co^2+^, 40.2% for Ca^2+^, 37.6% for Mn^2+^, and 33.7% for K^+^. At pH 9.5, Na^+^, Mg^2+^, and Co^2+^ induced modest but significant increases in caseinolytic activity (16.26%, 14.03%, and 5.02%, respectively), whereas Hg^2+^ (− 45%) and Fe^3+^ (− 36.24%) exerted strong negative modulation on enzyme activity (Fig. 3b).

### Activity of proteases toward different keratin substrates

**Fig. 4:**
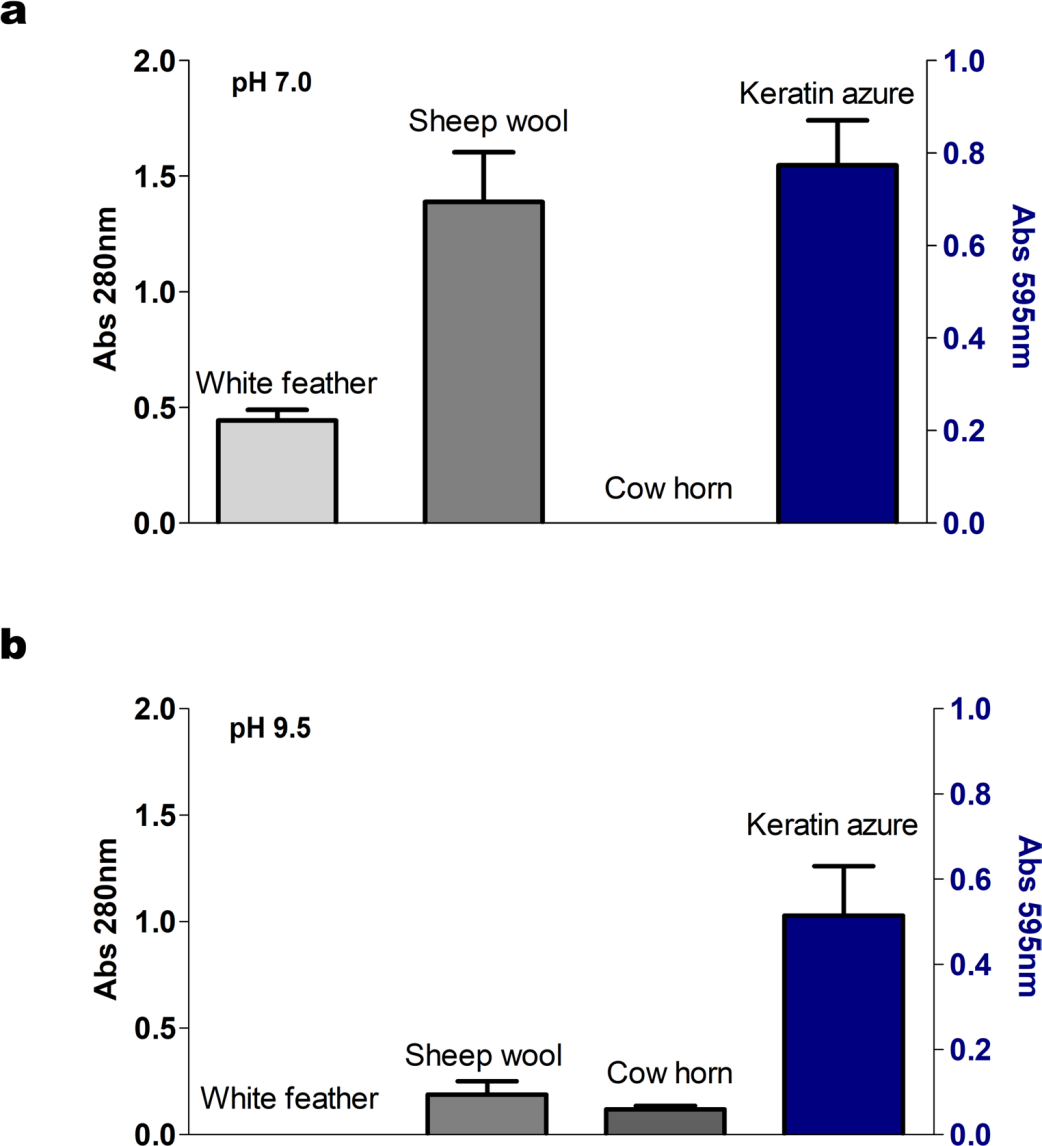
Action of proteases from the enzymatic extract of *B. subtilis* exposed to different sources of keratin at pH 7.0 (**a**) and pH 9.5 (**b**). Data are presented as mean ± SD (*n* = 3). Statistical significance was assessed by one-way ANOVA/Dunnett’s test (*p* < 0.05)

Proteases displayed differential capacities to hydrolyze different keratinous substrates. At pH 7.0, the enzymes demonstrated superior hydrolytic potential toward natural keratin sources than when tested at pH 9.5. These enzymes were effective in degrading white feathers and sheep wool, yielding mean absorbance values at 280 nm of 0.443 and 1.388, respectively (Fig. 4).

At pH 9.5, the proteolytic enzymes displayed limited but detectable ability to hydrolyze cow horn and sheep wool, yielding mean absorbance values of 0.118 and 0.180 at 280 nm, respectively. The activity toward azure keratin (absorbance at 595 nm of 0.509) was also lower than that of enzymes active at pH 7.0 (0.773).

## Discussion

Keratinases have emerged as promising biocatalysts for a variety of applications, including disruption of biofilms, generation of bioactive peptides, leather processing, and other industrial processes that require breakdown of protein materials.

Keratinases generally exhibit optimal activity under neutral to alkaline conditions, and most enzymes derived from mesophilic microorganisms display maximum catalytic activity between 40 and 60 °C [6]. Species of the genus Bacillus are among the most prominent microbial sources of extracellular proteases. Their ability to colonize diverse environments, with their broad metabolic repertoire, efficient secretion of enzymes, and high capacity to degrade protein-rich substrates [15] make these bacteria attractive targets for bioprospecting studies focused on keratinases.

Although *B. subtilis* has been extensively studied as a producer of proteases, the present study reports, for the first time, the diversity of its proteolytic enzymes and identifies at least seven well-defined proteases that exhibit activity against copolymerized collagen in polyacrylamide gels (Fig. 2c). These enzymes exhibit distinct mechanisms of action and their activities are differentially modulated by metal ions.

The catalytic mechanisms of these proteases can be inferred from their sensitivity to specific protease inhibitors. In the present study, the enzymatic activity of the crude extract was not significantly affected by cysteine protease inhibitors, such as iodoacetic acid and N-ethylmaleimide, indicating that cysteine-dependent catalysis does not play a significant role in this system. In contrast, substantial suppression of activity was observed in the presence of PMSF, a serine protease inhibitor, and EDTA, a metalloprotease inhibitor. This inhibitory profile suggests that the predominant enzymes in the extract are serine proteases and metalloproteases.

Metal ions represent a critical factor influencing protease activity, as they can contribute to both enzyme stabilization and catalytic efficiency [16]. Interactions between metal ions and amino acid functional groups can compromise protein folding and stability. Transition metals, such as Fe^3+^ and Cu^2+^, may be involved in redox reactions or form strong interactions with thiol, carboxyl, and amine groups, which can contribute to enzymatic inactivation. Hg^2+^ ions markedly reduced enzymatic activity under all evaluated conditions. Owing to its high affinity for thiol groups, Hg^2+^ can interact with cysteine thiols, forming stable complexes that disrupt native protein conformations necessary for catalysis. Such structural perturbations have been reported for other keratinolytic proteases exposed to Hg^2+^ [13].

Consistent with our results, Cu^2+^ has also been shown to substantially reduce the protease activity of various microbial species, including *Aphanoascus reticulisporus*, *Streptomyces coelicoflavus*, *Bacillus mycoides*, *Bacillus wiedmannii*, *Bacillus subtilis*, and *Bacillus altitudinis* [17]. At pH 9.5, Co^2+^ significantly increased keratinolytic activity, consistent with the observations of Lv et al. [18]

The enzymatic profile indicates that *B. subtilis* secretes multiple proteases during growth in liquid medium containing feathers, enabling the bacterium to efficiently utilize chicken feathers as a source of carbon and nitrogen. The ability to hydrolyze keratin, casein, and collagen (zymography analysis) demonstrates the functional versatility of the fermentation extract. Keratinolysis may be initiated by a specialized enzyme complex capable of breaking down the highly cross-linked keratin matrix. As keratin breakdown progresses and keratin-derived oligopeptides accumulate in the medium, additional proteases may subsequently act on these fragments, thereby contributing to the overall degradation process.

Proteases from *B. subtilis* have been shown to act on feathers, sheep’s wool, and horn materials, revealing the efficacy of enzymes in degrading α-keratins, whose structure is dominated by α-helices (sheep’s wool and horn), and β-keratins—dominated by β-sheets (feathers). Enzymes exhibiting collagenolytic, caseinolytic, and keratinolytic activities broaden the spectrum of application, enabling more comprehensive hydrolysis of protein-rich waste and increasing the efficiency of specialized processes, including wound debridement and waste management. The resulting protein hydrolysates may represent promising candidates for evaluation as biofertilizers and bioactive peptides (antioxidant activity, metal chelating agents, etc.) [6].

In other studies, keratinases from other microorganisms have demonstrated activity against insoluble keratinous substrates. Park et al. [19] demonstrated that a keratinase from *Pseudomonas geniculata* H10 effectively degraded feather meal, duck feathers, wool, hair, and nails. Revankar and Bagewadi [20] reported substantial keratinolytic activity by the enzyme extract of *Bacillus velezensis* ZBE1, which led to pronounced feather degradation, characterized by complete rupture of barbs and rachis within 48 h.

In summary, growing concerns over the environmental and health impacts associated with improper disposal of keratinous waste have intensified the search for effective biological solutions. *B. subtilis* is a promising microbial candidate for this purpose because it can efficiently degrade chicken feathers in submerged culture. The demonstrated ability of secreted proteases to initiate the degradation of highly resistant keratin substrates underscores their potential utility in processes aimed at valorization of protein-rich waste.

Further studies are needed to explore the full spectrum of applications for these enzymes, including optimization of fermentation conditions, enzyme engineering, and scalability assessments. Such efforts are essential to fully harness the potential of *B. subtilis* keratinases in sustainable industrial processes.

## Electronic Supplementary Material

Below is the link to the electronic supplementary material.

Supplementary Material 1
