# Supplementary material for: Keratinolytic proteases secreted by Bacillus subtilis obtained from feather-based submerged culture
Source: Braz J Microbiol. 2026 Jun 18;57(1):178. doi: 10.1007/s42770-026-01993-x (PMC13280098; doi:10.1007/s42770-026-01993-x)
Supplement: Supplementary file 1 — Supplementary Material 1 [file 42770_2026_1993_MOESM1_ESM.docx]

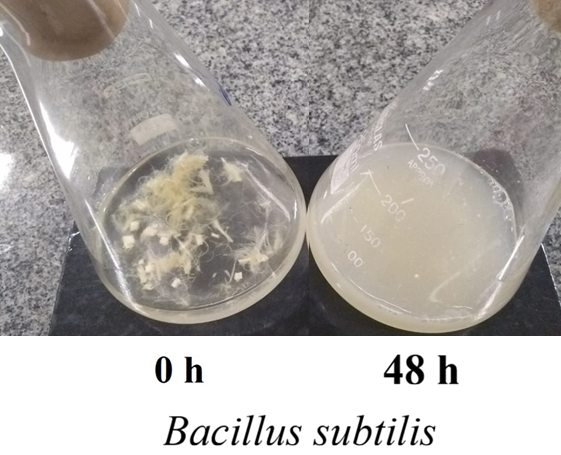
**Online Resource**

**Online Resource 1.** Feather solubilization by *Bacillus subtilis* during growth in a liquid medium containing 0.5% chicken feathers. At time zero (0 h), feathers were intact; after 48 h of cultivation, feather hydrolysate was observed.
